# Exploring the role of the core in sports performance: a systematic review of the effects of core muscle training

**DOI:** 10.3389/fspor.2025.1630584

**Published:** 2025-09-30

**Authors:** Juan Sebastian Bustos Carvajal, Florencio Arias Coronel

**Affiliations:** ^1^ Faculty of Education, University of Pamplona, Pamplona, Colombia; ^2^ Faculty of Health, Universidad Santiago de Cali, Cali, Colombia

**Keywords:** abdominal core, exercise, athletic performance, muscle strength, lower extremity

## Abstract

**Introduction:**

The Core or central area of the body, is a group of muscles in charge of providing stability and postural control, being crucial in sports performance. In sports, the practice of exercises to strengthen this region can improve different performance variables. However, most of these programs in athletes focus on direct and specific strengthening of the extremities, largely ignoring the role that this part of the body can have on strength and power. Therefore, the aim of this study is to systematically review the literature on the effects of core muscle training on key athletic performance parameters in competitive athletes, focusing primarily on core strength, dynamic balance, sprint speed, jump height, and agility.

**Materials and methods:**

A systematic review was carried out using a search strategy applied to Scopus, Science Direct, WebScience, Sportdiscuss and Sage Journal databases. Randomized con-trolled trials, quasi-experimental studies and prospective observational cohort studies evaluating the effect of core training on performance in competitive athletes participating at regional, national, or international levels were included.

**Results:**

Core training showed improvements in trunk extensor strength, core endurance, dynamic balance, and sprint speed in athletes. However, results regarding trunk flexor muscle strength, jump height and agility were not precise.

**Conclusion:**

In athletes, core training can improve core strength, dynamic balance, and sprint speed. However, its efficacy on other sports performan ce variables, such as agility or power, is not clear.

**Systematic Review Registration:**

https://www.crd.york.ac.uk/PROSPERO/view/CRD420251036787, identifier CRD420251036787.

## 1 Introduction

The Core or central area corresponds to the nucleus, or group of muscles located in the central part of the body. This muscle complex is crucial for athletic performance. This central area of the body, which encompasses the muscles of the abdomen, lower back, but-tocks, and pelvis, plays a critical role in several aspects of different sports. A strong core provides stability, improves the transfer of power from the legs to the pedals, maintains a proper posture on the bike, increases energy efficiency by allowing you to maintain an aerodynamic position for longer, prevents injuries by protecting the spine, improves balance. The muscles of the spine, trunk, abdomen and hips must be able to accelerate and decelerate the movements of the upper and lower limbs in a harmonic, coordinated and fluid synchrony (1). Maintaining a good stability of this complex depends mainly on an adequate interaction between the passive, active and neural subsystems (2). In this way, the core can be considered as a link and the center of almost all kinetic chains, playing an important role in maximizing the functions of the upper and lower extremities and athletic performance (3).

The importance of the function of the central core of the body for stabilization and force generation in all sports activities is increasingly recognized. “Core stability” is considered critical for efficient biomechanical function that maximizes force generation and minimizes joint loads in all types of activities, from running to throwing (4).

For example, in cycling the core muscles maintain the neutral pelvic position on the bike when both anterior and posterior muscle components are balanced (5). To prevent injury, proper bike adjustment is critical so that constant, low limb alignment is adopted throughout the ride (6). However, decreased core strength could artificially induce misalignment of the lower extremity in an effort to maintain a certain posture, while factors related to core stability may predict the risk of knee injuries (7, 8).

The impact of core strength training on lower limb power in athletes is an area that, to date, lacks sufficient conclusive studies. This gap in the scientific literature justifies the need for our research, which focuses on examining the relationship between core area muscle training and strength development in athletes. Therefore, the objective of this study is to provide a systematic review of the literature of core muscle training and its impact on athletic performance.

Traditionally, training to improve performance in sport has focused on the direct strengthening of the lower or upper limbs, prioritizing exercises such as squats, deadlifts and leg presses, among others also for upper limbs. Several studies have shown the effectiveness of these exercises to increase strength and power in the arms and legs. For example, Ronnestad et al. found significant improvements in the power of cyclists after a strength training program focused on the legs (9). Similarly, increases in maximum lower limb strength have been reported in cyclists after a period of weight training (10). However, emerging evidence suggests that a strong core is essential to optimize lower extremity function.

The effect of core training on leg strength and athletic performance is a topic that needs further investigation. Although there are some studies looking at how core stability affects athlete performance, there is little specific work on how core strengthening influences strength and power. This creates a lack of information in this field. Therefore, this research tries to fill this gap in the scientific literature, studying the relationship between the training of the core muscles and the increase of different variables such as speed, agility, power, among other variables of sports performance.

## 2 Materials and methods

This study was conducted following the recommendations of the Cochrane Collaboration and the PRISMA statement. El protocolo fue registrado en el registro prospectivo internacional de revisiones sistemáticas (PRÓSPERO): CRD420251036787.

The review focused on studies that included the following PICO structure: Population(P): Competitive athletes (between 18 and 65 years old, of both sexes) who regularly participated in competitions at the regional, national or international level. This broad age range was chosen to ensure a comprehensive search that would be inclusive of masters-level competitive athletes. Intervention (I): Core training programs (e.g., planks, stability exercises, Swiss ball training). Comparison (C): absence of core training, alternative workouts (such as limb-centered strength training), or control groups. Results (O): primary: core strength (measured by isometric or endurance tests such as plank time); secondary: dynamic balance (e.g., Star Excursion Balance Test), sprint speed (such as times in 10–20 m sprints), jump height (such as countermovement jumping), agility (e.g., Illinois Agility Test), and sport-specific performance (such as striking speed in soccer).

### 2.1 Inclusion criteria

We included randomized controlled trials, quasi-experimental studies (pre-post intervention with or without non-randomized control group) and prospective observational cohort studies evaluating the effect of core training on performance in athletes.

### 2.2 Exclusion criteria

Studies with participants who are not athletes (e.g., students, older adults, etc.); studies with interventions that do not include a core training program or that combine it with other interventions without an independent analysis of the effect of core training; interventions that focus solely on flexibility or stretching without a strengthening component; studies with methodological designs not suitable for a systematic review.

#### 2.2.1 Participants

Athletes (18–65 years old) of both sexes, competitive, defined by regular participation in competitions (regional, national or international).

#### 2.2.2 Factor to be evaluated

Analysis of Core musculature training protocols in athletes.

### 2.3 Primary result

#### 2.3.1 Sources of information

The literature search was conducted in the databases: Scopus, Science Direct, WebofScience, Sportdiscuss and Sage Journal, controlled clinical trials, case-control, cohort and cross-sectional studies were included from inception to December 2024, to ensure literature saturation, references of relevant articles identified through references, congresses, thesis databases, Open Grey, Google Scholar and Clinicaltrials.gov were scanned.

The search was performed using DeCS/Mesh terms and related words, the different combinations with the Boolean operators were used. No language or time restrictions were imposed.

### 2.4 Collect data

The information extraction process was carried out by two researchers, who reviewed each reference by title and summary. Subsequently, the full texts of the relevant studies were scanned, applying previously established inclusion and exclusion criteria, and extracting the relevant data. Any disagreement between the investigators was resolved by consensus.

Two trained reviewers used a standardized form to independently extract the following information from each article: study design, location, author names, title, objectives, number of patients included, study duration, outcome definitions, results obtained, and measures of association.

### 2.5 Risk of bias assessment (quality)

The quality of the studies and the risk of bias, in terms of clinical trials, the Pedro scale was applied, which examines randomization, blinding and the presentation of results. Studies that scored 4–6 on this scale.

## 3 Results

**Figure 1:**
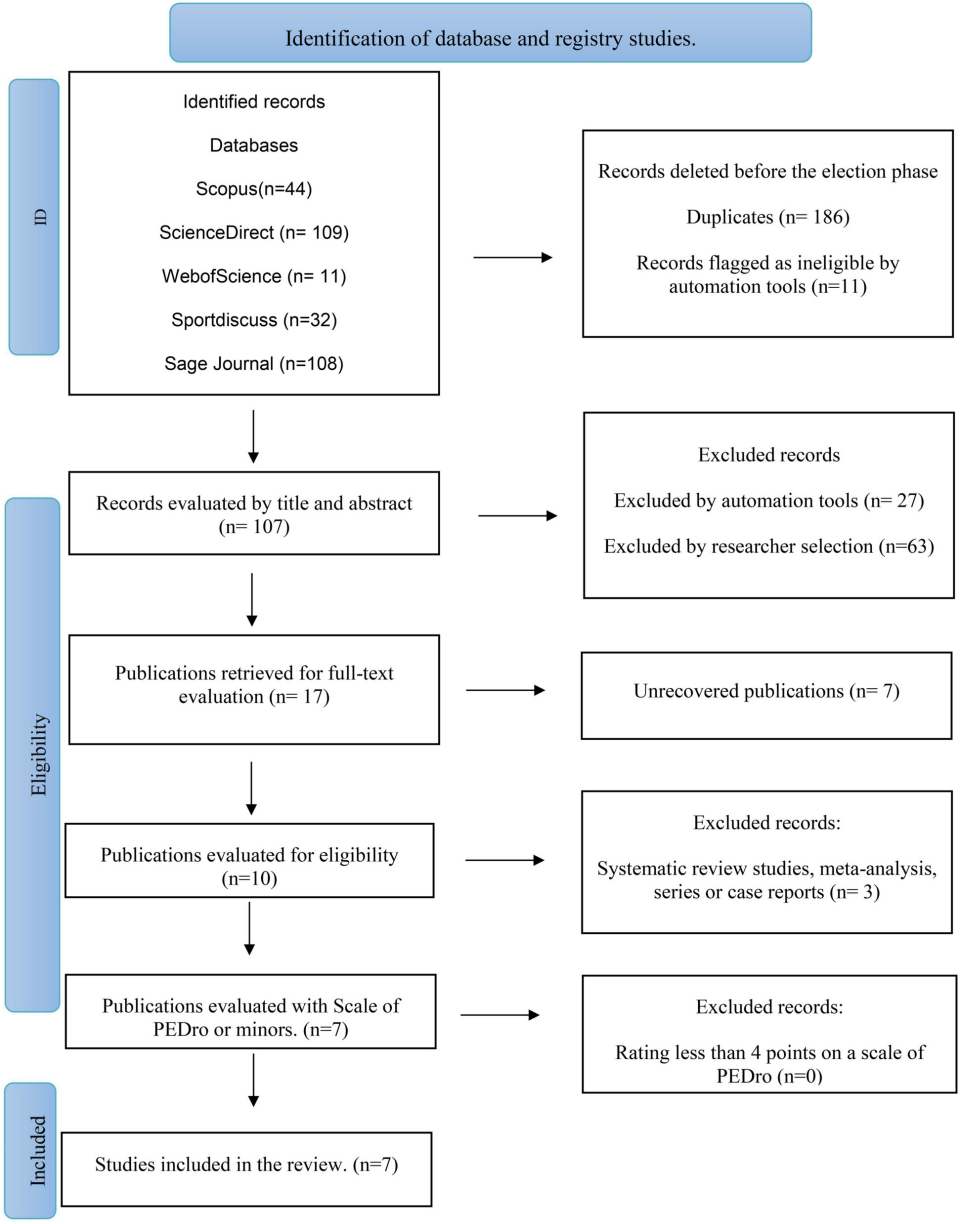
Flowchart of study selection process.

304 studies were initially identified from database searches. After excluding duplicate studies, 107 studies were evaluated by title and abstract, of which 90 were excluded be-because they were systematic reviews, letters to the editor, intervention protocols or because they were not related to the topic of interest. Subsequently, 17 full-text studies were analyzed, of which 7 did not meet the inclusion criteria, and finally, 7 studies were evaluated with the PEDro scale, which were finally included in this review (Figure 1).

### 3.1 Characteristics of included studies

7 articles were included, in their entirety they were of the controlled clinical trial type, 4 of the articles included were from the Asian continent (11–14), 2 from the European continent (15, 16) and 1 from the American continent (17): Taiwan, Turkey, Indonesia, China, Germany, Italy, and the United States, all of them in English (Table 1).

**Table 1:** Characteristics of the included studies.

Study (Settings)	Aim	Parameters of evaluated sports performance	Main results
Prieske, O., et al. Germany 2016 (15)	To investigate the effects of core strength training on stable versus unstable surfaces in combination with regular soccer training on trunk muscle strength/activation and athletic performance in young elite soccer players.	1. Core Muscle Strength/Activation 2. CMJ Jump Height 3. Sprint Time 4. Agility Time 5. Kick Performance	Improvements in trunk extensor strength, sprinting (10–20 m), and kicking were observed in both groups. No significant differences were found between groups (stable vs. unstable).
Tsai, Y.-J., et al. Taiwan 2020 (11)	To examine whether core training improves landing kinematics, athletic performance, and isokinetic strength in young volleyball players.	1. Trunk and lower extremity kinematics during landing. 2. Athletic performance (sprinting, agility, vertical jump). 3. Isokinetic hip and knee strength.	Decreased trunk flexion and knee internal rotation during landing. Increased isokinetic hip and knee strength. No significant changes in athletic performance.
Dello Iacono, A., et al. Italy 2014 (16)	To determine the effects of a core stability training program on balance control in soccer players.	1. Static equilibrium (COP). 2. Dynamic equilibrium (modified SEBT).	Improvements in static and dynamic balance in the intervention group.
Ozmen, T., & Aydogmus, M. Turkey 2016 (12)	To investigate the effect of core strength training on dynamic balance and agility in adolescent badminton players.	1. Dynamic balance (SEBT) 2. Agility (Illinois Agility Test), core endurance (AFT, BET, SBT).	Improvements in dynamic balance and core endurance were observed in the training group. No significant changes were observed in agility.
Amrullah, R., et al. Indonesia 2022 (13)	To determine the effectiveness of Swiss ball core stability training in Pencak Silat student-athletes.	1. Core strength (plank). 2. Dynamic balance (modified Bass test).	Improvements in core strength and dynamic balance in the experimental group.
Sato, K., & Mokha, M. United States 2009 (17)	To determine the effects of core strength training on running kinetics, lower extremity stability, and 5,000 m performance in runners.	1. GRF. 2. Stability (SEBT) 3. Performance at 5,000 m.	Improvement in 5,000 m time in the core training group. There were no changes in GRF or stability.
Liu Q., et al. China 2023 (14)	To investigate whether a core exercise program using slings can improve core endurance and basketball-specific performance in collegiate players.	1. Core endurance (FET, EET, RLET, LLET, 60-second sit-ups). 2. Basketball performance (vertical jump, free throws, set shots, obstacle layup).	The sling group improved core strength and hurdle layup time. No changes were observed in other performance measures.

CMJ, countermovement jump; COP, center of pressure; SEBT, star excursion balance test; AFT, anterior flexor test; BET, back extensor test; SBT, side bridge test; GRF, ground reaction force; FET, flexor endurance test; EET, extensor endurance test; RLET, right lateral endurance test; LLET, left lateral endurance test.

### 3.2 Characteristics of excluded studies

Excluded items showed no connection to the result of interest. In addition, letters to the editor, systematic/narrative reviews, medication intervention protocols were dispensed with.

### 3.3 Methodological quality

The average score on the PEDro scale for the studies analyzed, was 5 points with methodological quality studies, observing that the most frequent omissions were observed in the blinding of all therapists who administered the therapy and in the blinding of all evaluators who measured at least one key outcome.

### 3.4 Characteristics of the participants

Table 2 presents the characteristics of the participants, where it is observed that the samples varied between 8 and 30 participants. The age of the subjects included in the studies ranged from approximately 10–40 years. Regarding the sex of the participants, it was reported that in 3 of the studies it was carried out in males (11, 15, 16), 2 mixed (12, 17), and in 2 studies the sex was not specified (13, 14), thus being the male sex the most predominant. As for the sport practiced, in the studies it is mentioned that in 2 studies soccer (15, 16), 1 volleyball (11), 1 basketball (14), 1 badminton (12), 1 Pencak Silat (13), 1 career (16) are practiced. In addition, the practice time of the included athletes is known, which ranges from 1 to 10 years, however, in 4 of the studies this characteristic is not specified (13–15, 17).

**Table 2:** Characteristics of the intervention.

Study	Type of training	*n*	Age (Mean ± SD)	Sex (M/F)	Duration and frequency of training	Application parameters
Amrullah et al. (2022) (13)	Swiss ball-based core stability exercises	60 (30 experimental, 30 control)	15–17 years (mean and SD not specified)	Not specified	4 weeks, 3 times/week (experimental group)	Plank, Modified Bass Test (dynamic balance)
Sato and Mokha (2009) (17)	Core strength training (CST); control group without CST	20 (12 experimental, 8 control)	36.9 ± 9.4 years (initial screening: 28 participants)	M/F (10/18 initial screening)	6 weeks, 4 times/week (experimental group)	GRF (ground reaction forces), Star Excursion Balance Test, 5,000 m running time
Liu et al. (2023) (14)	Sling exercises (SET); control group without SET	40 (20 experimental, 20 control)	22 ± 4 years (experimental group); 22 ± 3 years (control group)	M/F (no ratio specified)	8 weeks, 2 times/week (experimental group)	FET, EET, LET (left and right), number of sit-ups in 60 s, vertical jump, shots (penalty and fixed position), lay-up over obstacles
Dello Iacono et al. (2014) (16)	Core Stability Training Program (CSTP); control group with regular warm-up	20 (10 experimental, 10 control)	18.5 ± 1.1 years	M	4 weeks, 5 times/week (experimental group)	COP (center of pressure) during one-legged stance with eyes open; modified Star Excursion Balance Test
Tsai et al. (2020) (11)	Core training; pre-post design with no control group	16	13.4 ± 1 years	M	6 weeks, 3 times/week	Trunk and lower limb kinematics during landing after jump and blocking, sports performance (10 m shuttle run, agility-T, vertical jump), isokinetic hip and knee strength.
Ozmen and Aydogmus (2016) (12)	Core strength training (CST); control group without CST	20 (10 experimental, 10 control)	10.8 ± 0.3 years	M/F (11/9)	6 weeks, 2 times/week (experimental group)	Star Excursion Balance Test, Illinois Agility Test, core resistance test (McGill)
Prieske et al. (2016) (15)	Core strength training on stable (CSTS) vs. unstable (CSTU) surfaces; both groups engaged in regular soccer training.	37 (19 CSTS, 18 CSTU; initial screening: 39 participants)	17 ± 1 year	M	9 weeks, 2–3 times/week (both groups)	Maximum isometric strength (MIS) of trunk flexors/extensors, CMJ height, 10–20 m sprint, agility (T-test), kick performance

GRF, ground reaction forces; SEBT, star excursion balance test; CST, core strength training; CSTP, core stability training program; COP, center of pressure; SET, sling exercise training; FET, flexor endurance test; EET, extensor endurance test; LET, lateral endurance test; CMJ, counter movement jump; CSTS, core strength training on stable surface; CSTU, core strength training on unstable surface; MIF, maximal isometric strength; MAV: mean average voltage.

### 3.5 Most evaluated sports performance parameters

The sports performance parameters most evaluated in the studies were core strength, dynamic balance and sprint speed. Core strength was measured primarily through the maximum isometric force (MIF) of trunk flexors and extensors, and core strength through endurance tests such as the plank test, abdominal fatigue test (aft), back extensor test (bet), and lateral bridge test (SBT). Dynamic balance was frequently assessed using the Star Excursion Balance Test (SEBT), analyzing range in different directions. Sprint speed was commonly measured over short distances (ex. 10–20 m). Other parameters such as the height of the countermovement jump (CMJ), agility (e.g., Agility Test T, Illinois Agility Test) and performance in specific sports gestures (e.g., football kicks) were evaluated less fre-quently, although their inclusion varies according to the specificity of the sport studied. Parameters such as power, local limb muscle endurance, flexibility, and aerobic endurance were reported to a lesser extent, suggesting a need for further research in these areas within the context of core training.

### 3.6 Results of the evaluated sports performance parameters

In the studies reviewed, core strength (Table 3), as measured through MIF, showed mixed results. While trunk extender MIF improved consistently after core training on both stable and unstable surfaces, flexor MIF showed no significant changes in most studies. Core endurance, as assessed by tests such as plank, aft, BET, and SBT, generally improved after training, with significant increases in endurance times.

**Table 3:** Core strength results.

Study	Cluster	Extent	Initial value	Final value	Difference/Tracking	*P* Value
Tsai et al. (2020) (11)	TG	Hip Flexors (N·m)	52.1	62.0	9.9	0.001*
		Hip Extensors (N m)	94.9	109.3	14.4	0.07
		Hip Abductors (N·m)	36.5	40.6	4.1	0.15
		Hip Adductors (N·m)	51.4	55.0	3.6	0.47
		Hip Internal Rotators (N m)	49.0	44.4	−4.6	0.18
		Hip External Rotators (N·m)	37.2	42.8	5.6	0.04
		Knee Flexors (N·m)	65.5	80.8	15.3	0.02
		Knee Extensors (N·m)	86.1	107.0	20.9	0.003*
Liu et al. (2023) (14)	TG	(FET) (sec)	60.0 ± 16.4	226.4 ± 106.7	+166.4	<0.01*
		(EET) (sec)	115.9 ± 23.2	157.7 ± 35.9	+41.8	<0.01*
		(LLET) (sec)	100.1 ± 27.3	142.3 + 34.9	+42.2	<0.01*
		(RLET) (sec)	109.1 ± 41.8	154.1 ± 36.8	+45.0	<0.01*
		Number of Sit-ups in 60 s	38.8 ± 3.5	51.1 ± 4.1	+12.3	<0.01*
	GC	(FET) (sec)	50.9 ± 12.3	119.5 ± 40.9	+68.6	<0.01*
		(EET) (sec)	112.7 ± 25.9	120.9 ± 26.8	+8.2	NS
		(LLET) (sec)	112.3 ± 31.8	101.3 ± 33.2	−11	NS
		(RLET) (sec)	104.1 ± 34.9	93.6 ± 29.1	−10.5	NS
		Number of Sit-ups in 60 s	40.5 ± 3.4	44.0 ± 2.3	+3.5	NS
Ozmen & Aydogmus (2016) (12)	TG	(AFT) (sec)	42.17 + 29.21	107.52 ± 22.54	+65.35	0.00*
		(BET) (sec)	43.90 + 34.28	90.70 ± 7.22	+46.80	0.004*
		(SBT) (sec)	24.44 ± 11.17	52.24 ± 7.10	+27.80	0.003*
	GC	(AFT) (sec)	39.13 + 25.14	43.23 ± 23.43	+4.10	NS
		(BET) (sec)	34.20 ± 33.57	39.41 ± 36.72	+5.21	NS
		(SBT) (sec)	17.34 ± 12.60	22.68 ± 14.92	+5.34	NS
Prieske et al. (2016) (15)	CSTS	MIF Trunk Flexors (N)	656.5 ± 92.3	681.0 ± 89.3	+3.7%	0.47 (NS)
		MIF Trunk Extensors (N)	603.1 ± 98.8	644.0 + 92.6	+6.8%	0.02*
		MAV Flexors (%)	47.9 ± 9.5	56.8 ± 4.4	+18.4%	0.11 (NS)
		MAV Extensors (%)	60.5 ± 4.9	62.3 ± 5.4	+3.0%	0.83 (NS)
	CSTU	MIF Trunk Flexors (N)	624.4 ± 99.6	617.7 ± 97.6	−1.1%	0.47 (NS)
		MIF Trunk Extensors (N)	591.4 ± 67.1	614.0 ± 115.1	+3.8%	0.02*
		MAV Flexors (%)	53.2 ± 11.4	55.6 ± 12.7	+4.6%	0.11 (NS)
		MAV Extensors (%)	63.2 ± 3.2	62.4 ± 6.0	−1.3%	0.83 (NS)
Amrullah et al. (2022) (13)	TG	Plank test (sec)	18.630	27.683	+0.9053	0.000*
	GC	Plank test (sec)	17.857	21.673	+0.3817	0.000*

N·m, newton meter; sec, seconds; FET, flexor endurance test; EET, extender endurance test; LLET, left lateral endurance test; RLET, right lateral endurance test; AFT, abdominal fatigue test; BET, back-extensor test; SBT, side bridge test; MIF, maximum isometric force; MAV, mean amplitude value; NS, not significant; (N), newtons; TG, training group; CG, control group; CSTS, core strength training on stable surface; CSTU, core strength training on unstable surface.

*Statistical significance in relation to the *P*-values reported in the analyzed studies.

Studies that used SEBT to assess dynamic balance showed significant improvements in range in all three directions assessed (anterior, posteromedial, and posterolateral), with greater improvements often observed in the group that trained with unstable surfaces (Table 4).

**Table 4:** Balance results.

Study	Cluster	Extent	Initial value	Final value	Difference/Tracking	*P* Value
Dello Iacono et al. (2014) (16)	TG	AP Range (mm) RL	6.9 ± 1.6	5.8 ± 1.2	−1.1	0.021*
		Range ML (mm) RL	8.4 ± 2.7	8.3 ± 4.9	−0.1	0.567
		FROM AP (mm) RL	0.62 ± 0.11	0.33 ± 0.01	−0.29	<0.001*
		FROM ML (mm) RL	0.88 ± 0.13	0.81 ± 0.06	−0.07	0.882
		OV AP (mm/s) RL	17.4 ± 1.1	12.6 ± 0.1	−4.8	0.037*
		OV ML (mm/s) RL	16.9 ± 0.8	17 ± 0.9	0.1	0.783
		FC AP (Hz) RL	10.2 ± 0.7	10.3 ± 0.9	0.1	0.116
		Centroid Frequency ML (Hz) RL	9.98 ± 0.9	9.89 ± 0.8	−0.09	0.446
		AP Range (mm) LL	6.9 ± 1.7	5.8 ± 1.8	−1.1	0.026*
		Range ML (mm) LL	5.4 ± 0.1	4.8 ± 0.1	−0.6	0.033*
		FROM AP (mm) LL	0.6 ± 0.01	0.4 ± 0.17	−0.2	0.017*
		FROM ML (mm) LL	0.67 ± 0.21	0.32 ± 0.06	−0.35	0.014*
		OV AP (mm/s) LL	14.2 ± 1.1	11.6 ± 0.1	−2.6	0.041*
		OV ML (mm/s) LL	18.3 ± 0.4	15.1 ± 0.2	−3.2	0.031*
		FC AP (Hz) LL	9.88 ± 0.6	10.22 ± 0.7	0.34	0.036*
		FC ML (Hz) LL	9.01 ± 0.9	9.51 ± 0.9	0.5	0.044*
		SEBT MADX (%) RL	107.9 ± 10.3	114.2 ± 9.1	+6.3	0.03*
		SEBT MADX (%) LL	111.7 ± 9.1	115.5 ± 8.6	+3.8	0.04*
		A. Anterior (cm)	4.9 ± 4.2	2.1 ± 2.4	−2.8	0.013*
		A. Posteromedial (cm)	3.3 ± 0.8	2.8 ± 2.1	−0.5	0.145
		A. Posterolateral (cm)	1.8 ± 0.6	1.1 ± 2.1	−0.7	0.234
	CG	AP Range (mm) RL	5.6 ± 1.2	4.4 ± 2.1	−1.2	0.015*
		Range ML (mm) RL	5.2 ± 1.7	5.3 ± 0.7	0.1	0.752
		FROM AP (mm) RL	0.67 ± 0.02	0.32 + 0.06	−0.35	0.041*
		FROM ML (mm) RL	0.59 ± 0.12	0.58 ± 0.16	−0.01	0.674
		OV AP (mm/s) RL	21.3 ± 0.7	16.5 ± 1.2	−4.8	0.029*
		OV ML (mm/s) RL	20.3 ± 1.2	18.9 ± 1.7	−1.4	0.134
		FC AP (Hz) RL	10.2 ± 0.8	10.0 ± 0.98	−0.2	0.029*
		FC ML (Hz) RL	9.48 ± 0.8	9.4 ± 1.3	−0.08	0.123
		AP Range (mm) LL	8.6 ± 0.2	8.6 ± 0.1	0	0.877
		Range ML (mm) LL	4.6 ± 0.1	5.1 ± 0.1	0.5	0.08
		FROM AP (mm) LL	0.68 ± 0.03	0.68 ± 0.06	0	0.882
		FROM ML (mm) LL	0.59 ± 0.12	0.58 ± 0.16	−0.01	0.674
		OV AP (mm/s) LL	15.9 ± 0.8	16.1 ± 0.9	0.2	0.883
		OV ML (mm/s) LL	19.3 ± 0.2	19.9 ± 1.1	0.6	0.234
		FC AP (Hz) LL	9.77 ± 0.9	9.65 ± 1	−0.12	0.146
		FC ML (Hz) LL	9.88 ± 1.1	9.78 ± 1.1	−0.1	0.101
		SEBT MADX (%) RL	99.2 ± 12.5	99.2 ± 12.6	0	0.75
		SEBT MADX (%) LL	101.5 ± 9.9	104.5 ± 13.1	3	0.06
		A. Anterior (cm)	4.9 ± 5.2	6.8 ± 7.4	+1.9	0.181
		A. Posteromedial (cm)	5.2 ± 3.2	4.8 ± 3.7	−0.4	0.169
		A. Posterolateral (cm)	1.7 ± 2.7	1.7 ± 2.3	0	0.668
Sato & Mokha (2009) (17)	TG	SEBT (% Leg Length)	198.75	220.67	+21.92	NS
	CG	SEBT (% Leg Length)	199.13	209.38	+10.25	NS
Amrullah et al. (2022) (13)	TG	Modified Bass Balance Test	266.667	350.000	83.333	0.000*
	CG	Modified Bass Balance Test	220.000	268.333	48.333	0.000*
Ozmen & Aydogmus (2016) (12)	TG	Previous SEBT (A) (%)	85.07 ± 5.42	92.54 ± 6.44	+7.47	0.00*
		Posteromedial (PM) SEBT (%)	97.19 ± 4.36	102.88 ± 4.16	+5.69	0.00*
		Posterolateral (PL) SEBT (%)	91.20 ± 5.65	95.98 ± 5.36	+4.78	0.00*
	CG	Previous SEBT (A) (%)	80.75 ± 8.10	82.53 ± 7.70	+1.78	0.00*
		Posteromedial (PM) SEBT (%)	91.86 ± 5.28	93.01 ± 5.44	+1.15	0.00*
		Posterolateral (PL) SEBT (%)	87.48 ± 6.09	88.90 ± 6.39	+1.42	0.00*

mm, millimeters; SD, standard deviation; OV, oscillation velocity; CF, centroid frequency; RL, right leg; LL, left leg; Hz, Hertz; SEBT, star excursion balance test; MADX, SEBT composite score; A, previous; PM, posteromedial; PL, posterolateral; LL, limb length; cm, centimeters; TG, training group; CG, control group; NS, not significant.

*Statistical significance in relation to the *P*-values reported in the analyzed studies.

In terms of sprint speed (Table 5), most studies reported significant improvements, especially in short sprints (ex. 10–20 m), regardless of the type of core training. However, the results for CMJ height were inconsistent, with some studies showing improvements and others showing no significant changes.

**Table 5:** Sprint speed results.

Study	Cluster	Extent	Initial value	Final value	Difference/Tracking	*P* Value
Tsai et al. (2020) (11)	Intervention	Shuttle Race (s)	5.7	5.5	−0.2	0.11 (NS)
Prieske et al. (2016) (15)	CSTS	Sprint time 0–10 m (s)	1.69 ± 0.04	1.72 ± 0.08	+1.9%	0.10 (NS)
		Sprint time 10–20 m (s)	1.27 ± 0.02	1.22 ± 0.04	−3.6%	<0.001*
		Sprint time 0–20 m (s)	2.96 ± 0.05	2.95 ± 0.11	−0.5%	0.52 (NS)
	CSTU	Sprint time 0–10 m (s)	1.71 ± 0.06	1.73 ± 0.06	+1.3%	0.10 (NS)
		Sprint time 10–20 m (s)	1.28 ± 0.05	1.25 ± 0.02	−2.7%	<0.001*
		Sprint time 0–20 m (s)	2.99 ± 0.11	2.97 ± 0.07	−0.4%	0.52 (NS)

s, seconds; CSTS, core strength training on stable surface; CSTU, core strength training on unstable surface; NS, not significant.

*Statistical significance in relation to the *P*-values reported in the analyzed studies.

Agility (Table 6), measured through tests such as Agility T or Illinois, showed no solid improvements after core training in most studies, suggesting that this type of training may not transfer benefits directly to agility.

**Table 6:** Agility results.

Study	Cluster	Extent	Initial value	Final value	Difference/Tracking	*P* value
Tsai et al. (2020) (11)	Intervention	Agility Test T (s)	11.8	11.1	−0.7	0.07*
Ozmen & Aydogmus (2016) (12)	Training	IAT(s)	23.12 ± 2.05	20.43 ± 1.93	−2.69	0.16 (NS)
	Control	IAT(s)	23.79 ± 1.22	22.05 ± 1.48	−1.74	0.16 (NS)
Prieske et al. (2016) (15)	CSTS	Agility Test Time T (s)	9.7 ± 0.4	9.7 ± 0.5	−0.2%	0.83 (NS)
	CSTU	Agility Test Time T (s)	9.7 ± 0.3	9.7 ± 0.4	+0.6%	0.83 (NS)

s, seconds; IAT, illinois agility test; NS, not significant; CSTS, core strength training on stable surface; CSTU, core strength training on unstable surface.

*Statistical significance in relation to the *P*-values reported in the analyzed studies.

Finally, in studies that evaluated specific sports performance (Table 7), such as kick speed in soccer, improvements were observed after core training, but the magnitude of these improvements and their statistical significance varied between the studies analyzed.

**Table 7:** Sports performance results.

Study	Cluster	Extent	Initial value	Final value	Difference/Tracking	*P* value
Tsai et al. (2020) (11)	TG	CMJ	43.1	44.8	1.7	0.63
Liu et al. (2023) (14)	TG	Free Throw (Hits/20)	11.1 ± 2.3	13.8 ± 2.1	+2.7	NS
		Fixed Position Shooting (Hits/20)	7.6 ± 3.4	10.2 ± 1.7	+2.6	NS
		Vertical Jump and Reach (m)	0.76 ± 0.1	0.77 ± 0.1	+0.01	NS
		Obstacle Course Time with Tray (sec)	21.0 ± 2.5	16.1 ± 0.8	−4.9	<0.01*
	CG	Free Throw (Hits/20)	11.0 ± 3.4	12.9 ± 3.2	+1.9	NS
		Fixed Position Shooting (Hits/20)	6.2 ± 3.2	9.6 ± 2.7	+3.4	NS
		Vertical Jump and Reach (m)	0.79 ± 0.2	0.81 ± 0.2	+0.02	NS
		Time on Obstacle Course with Tray (s)	20.2 ± 3.3	18.8 ± 3.2	−1.4	NS
Sato & Mokha (2009) (17)	TG	5,000 m race time (min:s)	29:29:00	28:42:00	−0:47	0.05*
	CG	5,000 m race time (min:s)	26:30:00	26:13:00	−0:17	0.05*
Prieske et al. (2016) (15)	CSTS	Countermovement Jump Height (CMJ) (cm)	36.0 ± 3.4	35.5 ± 3.2	−1.5%	0.82 (NS)
		Kick Performance (km/h)	107.9 ± 5.8	107.5 ± 6.1	−0.3%	0.003*
	CSTU	CMJ Height (cm)	34.0 ± 3.4	34.3 ± 2.7	+0.7%	0.82 (NS)
		Kick Performance (km/h)	101.3 ± 6.8	103.4 ± 6.3	+2.1%	0.003*

cm, centimeters; s, seconds; N·m, newton meter; km/h, kilometers per hour; NS, not significant; CSTS, core strength training on stable surface; CSTU, core strength training on unstable surface; CMJ, counter movement jump; TG, training group; CG, control group.

*Statistical significance in relation to the *P*-values reported in the analyzed studies.

## 4 Discussion

This systematic review analyzed the effect of core strength training on athletic performance in athletes of different practices. After an exhaustive literature search, 7 studies (11–17), were identified which met the inclusion criteria, evaluated with the PEDro scale (mean score of 5 points). The studies, predominantly controlled clinical trials, covered various sports disciplines (football, volleyball, basketball, badminton, Pencak Silat and running) and geographical contexts (Asia, Europe and America). The total sample of participants, aged between 10 and 40 years, was predominantly male. The considerable heterogeneity in training methodologies, which encompassed variations in duration (ranging from 4 to 9 weeks), frequency, and modalities (e.g., Swiss ball, slings, stable and/or unstable surfaces) together with the diversity of performance parameters assessed, constitutes an inherent limitation for evidence synthesis and adds complexity to the interpretation and generalization of findings. This variability may have masked specific effects or contributed to the inconsistency of certain outcomes, underscoring the difficulty of establishing optimal and universally applicable core training protocols based solely on the current body of studies.

The most researched variables were core strength, dynamic balance, and sprint speed. Core strength, measured through the maximum isometric force (MIF) of trunk flexors and extensors, showed dissimilar results. While extender MIF improved consistently after training, as reported by Prieske et al. (15), in elite young footballers, flexor MIF did not experience significant changes in most studies, consistent with the observations of Ozmen and Aydogmus (12), in badminton players. This discrepancy could be attributed to the greater involvement of trunk extensors in postural stabilization and force transfer during sports practice (4). In contrast, the results for maximal isometric trunk flexor strength (MIF) were less conclusive, with most studies reporting no significant changes. This discrepancy, compared with the improvements observed in the extensors, may be due to the fact that the reviewed core training protocols did not sufficiently emphasize maximal loading of the flexors, or that the nature of the exercises prioritized stability and endurance over maximal strength in this direction. It is possible that the neural and structural mechanisms involved in isokinetic flexor strength require different or more specific training stimuli than those implemented in the included studies. Core endurance, as assessed by plank, aft, BET, and SBT, improved after the intervention, showing an increase in endurance times. This result is similar to different previous studies demonstrating the efficacy of core training to improve the endurance of the trunk musculature (18). Similarly, research has been conducted on core stability exercises that strengthen the abdominal, lower back, and pelvic muscles, improving stability and control during the throwing motion (19).

Dynamic balance, assessed primarily with SEBT, showed significant improvements in all three axes of movement (anterior, posteromedial, and posterolateral), particularly in the group that trained on unstable surfaces (12). However, it is important to consider that the magnitude of the improvement in balance may vary according to the level of previous training of the athletes, as suggested by Granacher et al. (20), in their study with adolescents. In terms of sprint speed, most studies, including that of Prieske et al. (15), showed significant improvements, especially over short distances. This could be explained by the greater stability of the core, which allows a more efficient transfer of force to the lower extremities during the stroke (21).

Similarly, the results for countermovement jump (CMJ) height were inconsistent, with some studies reporting improvements while others did not. The CMJ is a multifactorial action that largely depends on lower-limb explosive strength, intermuscular coordination, and jump technique (22). Core training, while essential for providing a stable base, is unlikely to be the primary limiting factor for CMJ performance in athletes; rather, its impact may be more indirect and contingent upon integration with plyometric and lower-limb strength training. With respect to agility, as assessed through tests such as the T-Test or Illinois Agility Test, the results were particularly weak and showed no consistent improvements in most studies following core training (12, 15). This finding suggests a limited transfer from isolated core training to agility. Agility is a complex skill that not only requires trunk stability and strength but also cognitive components (perception and decision-making), rapid changes of direction, acceleration and deceleration, and braking ability. While a strong core provides a foundation, specific agility training that integrates these elements in sport-related contexts is essential to achieve meaningful improvements (23, 24). Therefore, core training may be more effective as a foundational component that prepares athletes for more specific and functional agility programs, rather than serving as a substitute for them.

Finally, in studies that evaluated specific sports performance, such as kick speed in soccer (14), improvements were observed after core training. However, the magnitude of these findings and their statistical significance were variable. This reinforces the importance of the principle of specificity in training, where adaptations are specific to the type of activity performed (25), also as Hawley points out, which indicates that this principle predicts that the closer the training routine comes to the requirements of the desired result (i.e., a specific exercise task or performance criteria), the better the result (26).

In summary, core training seems to have a positive effect on the different variables studied such as the strength of the trunk extenders, core resistance, dynamic balance and sprint speed.

Finally, it is imperative to acknowledge the inherent limitations of the included studies that affect the reliability of our findings. Most trials involved relatively small sample cohorts (ranging from 8 to 30 participants), which reduces statistical power and limits the generalizability of the results to broader athletic populations. Furthermore, while our inclusion criteria encompassed a broad age range (18–65 years) to potentially include masters athletes, the literature search only yielded studies with participants up to 40 years of age. Consequently, our findings cannot be generalized to older competitive athletes, and the effects of core training in that specific demographic remain an area for future investigation. Moreover, methodological quality, as assessed with the PEDro scale (average score of 5), revealed frequent omissions in therapist and assessor blinding. The lack of therapist blinding introduces a risk of performance bias, while the absence of assessor blinding may lead to detection bias, whereby the expectations of researchers or participants influence the reported outcomes. These methodological factors must be critically considered when interpreting the magnitude and consistency of the effects of core training.

## 5 Conclusions

Despite the limitations to which this review was subjected, due to the number of studies selected and the heterogeneity in the methods used by them, the findings suggest that core musculature training could constitute a valuable tool for improving certain performance parameters in sport. Overall, there is a trend toward improvement in trunk extension strength, core endurance, dynamic balance, and sprint speed in athletes who added core training to their training protocols. However, results regarding trunk flexor strength, CMJ height, and agility were inconclusive. These results have scientific and clinical implications. From a scientific perspective, it is proposed that future research adopt more rigorous methodological control, including appropriately calculated sample sizes to ensure statistical power and representativeness. Prioritizing assessor blinding and, whenever ethically feasible, participant or therapist blinding is crucial to minimize the risk of detection and performance bias. More homogeneous training protocols are recommended; alternatively, any heterogeneity should be explicitly justified and discussed to enhance understanding of the specific relationship between core training and components of sports performance. Future research should also explore designs that allow for the analysis of interactions between core training and other training modalities (e.g., plyometric, agility-specific), in order to better elucidate how the core contributes to holistic and functional athletic performance. From a clinical perspective, these preliminary results suggest that exercises that focus on the core area would be beneficial for athletes in various sports, particularly those that focus on endurance and dynamic stability. Contributing in this way to the improvement of strength, speed and balance. Even so, it is necessary to act with caution when generalizing these findings, due to the limitations presented and the variability in the individual response to training. Sports and health professionals are encouraged to take into account the specificity of each sport and the individual needs of athletes when designing and implementing core area muscle training programs.

## Data Availability

The original contributions presented in the study are included in the article/Supplementary Material, further inquiries can be directed to the corresponding author.
