# Supplementary material for: Exploring the role of the core in sports performance: a systematic review of the effects of core muscle training
Source: Front Sports Act Living. 2025 Sep 30;7:1630584. doi: 10.3389/fspor.2025.1630584 (PMC12518322; doi:10.3389/fspor.2025.1630584)
Supplement: Supplementary file 1 [file Table1.docx]

| **Appendix A**. Search equations | | | | | | | | | | |
| --- | --- | --- | --- | --- | --- | --- | --- | --- | --- | --- |
| Abdominal core | AND | exercise | OR | Bicycling | AND | Athletic Performance | AND | Muscle Strength | OR | Lower Extremity |
| Abdominal Core | AND | exercise | AND | Athletic Performance | AND | Bicycling | AND | Muscle Strength | AND | Lower Extremity |
| Athletic Performance | AND | Muscle Strength | AND | Lower Extremity | AND | Core |  |  |  |  |
| core strength | AND | exercise | AND | Athletic Performance | AND | Muscle Strength | OR | Bicycling | AND | Lower Extremity |
| Abdominal Core | AND | exercise | AND | Athletic Performance | OR | Bicycling | AND | Muscle Strength | AND | Lower Extremity |
